# Quality of postpartum family planning services in Jampersal patients in sub-district of Panjatan, Kulon Progo

**DOI:** 10.1186/1471-2458-14-S1-P21

**Published:** 2014-01-29

**Authors:** Rina Nuryati, Mubasysyr Hasanbasri, Muhammad Hakimi

**Affiliations:** 1 Health Policy and Management, Universitas Gadjah Mada, Yogyakarta 55281, Indonesia; 2 Department of Health Policy and Management, Universitas Gadjah Mada, Yogyakarta 55281, Indonesia; 3 Department of Maternal and Child Health, Universitas Gadjah Mada, Yogyakarta 55281, Indonesia

## Background

Jampersal (jaminan persalinan or insurance for delivery) is one of the programmes of the Ministry of Health to accelerate the MDG’s 4 and 5 to eliminate financial barriers and increase access to safe health and delivery, high quality and affordable cares. Jampersal service package includes antenatal care, childbirth, postnatal care, and family planning. It is also expected that birth spacing or the incidents of “4 too”-- too old, too young, too much, and too close, were monitored. The purpose of this study was to view the quality of postpartum family planning and whether it has reached the optimum output.

## Materials and methods

This study was a qualitative research using a case study design. This study was conducted in sub-district of Panjatan, Kulon Progo, Yogyakarta. The respondents were patients who used Jampersal Insurance in their 42 days postpartum periods, midwives, field officers, health centre’s head, and district health office staff.

## Results

Of all Jampersal patients in 2012, only 28.37% accepted postpartum family planning. Yet the implementation of quality counselling from the midwives led to patients still being undecided about postpartum family planning options. Moreover, the regional health insurance system (Jamkesda) also created confusion because an insurance scheme for delivery for the poor had been in place. Without counselling for family planning, Jamkesda was more popular than Jampersal.

## Conclusions

Acceptance of postpartum family planning using Jampersal scheme was related to the counselling given by the midwives. Policy support at the local level is needed to be in line with the central policy.

